# Artificial reefs reduce the adverse effects of mud and transport stress on behaviors of the sea cucumber *Apostichopus japonicus*

**DOI:** 10.1038/s41598-023-36791-0

**Published:** 2023-06-13

**Authors:** Fangyuan Hu, Huiyan Wang, Ruihuan Tian, Guo Wu, Luo Wang, Yaqing Chang, Chong Zhao

**Affiliations:** Key Laboratory of Mariculture & Stock Enhancement in North China’s Sea, Ministry of Agriculture and Rural Affairs, Dalian Ocean University, Dalian, 116023 China

**Keywords:** Fisheries, Marine biology, Behavioural ecology

## Abstract

Poor survival of seeds reduces the production efficiency of the sea cucumber *Apostichopus japonicus* in pond culture. We investigated the effects of sea mud on the movement-related behaviors of *A*. *japonicus* with different body sizes. Mud significantly decreased crawling behavior and wall-reaching behavior in small seeds (~ 1 g of body weight), but not in the large ones (~ 2.5 g of body weight). These behaviors were significantly greater in the large seeds of *A*. *japonicus* than those in the small individuals when they were both on the mud. This clearly suggests that mud has negative effects on the movement-related behaviors of small seeds, but not on large individuals. We further assessed the effects of inevitable transport stress on the movement-related behaviors of *A*. *japonicus* on mud. Significantly poorer performances in crawling behavior, wall-reaching behavior and struggling behavior were observed in stressed *A*. *japonicus* (both sizes) than those in unstressed groups*.* These new findings indicate that transport stress further increases the adverse effects on the movement-related behaviors of *A*. *japonicus* on mud. Moreover, we investigated whether adverse effects can be reduced when individuals are directly seeded onto artificial reefs. Crawling behavior, wall-reaching behavior and struggling behavior in stressed *A*. *japonicus* (both sizes) seeded onto artificial reefs were significantly greater than those on mud, whereas artificial reefs did not significantly improve the crawling and struggling behaviors of unstressed small seeds. These results collectively indicate that mud and transport stress show negative impacts on the movement-related behaviors of sea cucumbers. Artificial reefs greatly reduce these adverse effects and probably contribute to improving the production efficiency of sea cucumbers in pond culture.

## Introduction

Aquaculture plays an essential role in providing food and nutrition. Production from aquaculture and fisheries reached 214 million tonnes with a value of USD 424 billion^1^. In China, sea cucumbers *Apostichopus japonicus* are becoming more popular recent years because of their high medicinal and nutritional values^2^, compared to other cultured species. The increasing market demands greatly stimulate the aquaculture of *A*. *japonicus*, with an annual production of 222,707 metric tons in 2021 in China^3^. Pond culture plays a leading role in producing sea cucumbers^4^. The culture area of ponds, for example, is 425,522 hectares in 2021 in China^3^. Improving production efficiency is essential in aquaculture. Seed mortality, however, greatly reduces the production efficiency in the pond. Almost 100% of small seeds of sea cucumber (1 g of wet weight) died when they were directly seeded into the pond in Dalian^5^. This indicates body size is an important factor affecting the survival of seeds in pond culture^6^. Large seeds (> 2.5 g of wet weight) are commonly used in pond culture because of better survival. Small seeds (< 1 g of wet weight) are kept at the hatchery for ~ 3 months until reaching the appropriate size^6^. However, large seeds are costly to reach the same seeding net biomass compare with small seeds, which directly decreases the economic benefits. It is important to know the reasons for the mortality of seeds and accordingly establish an effective method to improve the production efficiency of sea cucumbers in pond culture.

The substrate in aquaculture pond is mainly silt which is difficult for sea cucumbers to crawl, because they cannot attach to the mud substrate^7^. Sea cucumbers were seldom found in muddy habitats in the field, despite the abundance of organic matter^8,9^. Therefore, it is reasonable to speculate that sea cucumbers probably get stuck in the mud and may consequently lead to death. Further, sea cucumbers are inevitably exposed to stress (e.g. handling and anoxia stresses) when they are transported from the seed hatchery to a pond. Environmental stress adversely affects the movement-related behaviors of sea cucumbers. For example, small *A*. *japonicus* exposed to high-intensity handling stresses showed decreased movement and foraging behaviors^10^. Thus, transport stress probably increases the adverse effects on the movement-related behaviors of sea cucumbers on mud. Juvenile sea cucumbers choose suitable habitats in the field for better fitness^11,12^. It has been well documented that artificial reefs (e.g. oyster shells and tiles) greatly improve the aggregation behavior of sea cucumbers^13,14^. Therefore, it is worth investigating whether directly seeding sea cucumbers onto artificial reefs reduces the adverse effects.

The purpose of the present study is to investigate the behavioral responses of *A*. *japonicus* to mud under various conditions, which may provide valuable information for pond culture. We ask (1) whether mud adversely affects the movement-related behaviors of *A*. *japonicus* in different body sizes; (2) whether transport stress increases the adverse effects on the movement-related behaviors on mud; (3) whether adverse effects can be reduced by seeding individuals onto the artificial reefs.

## Results

### Experiment I

#### Crawling behavior

**Figure 1:**
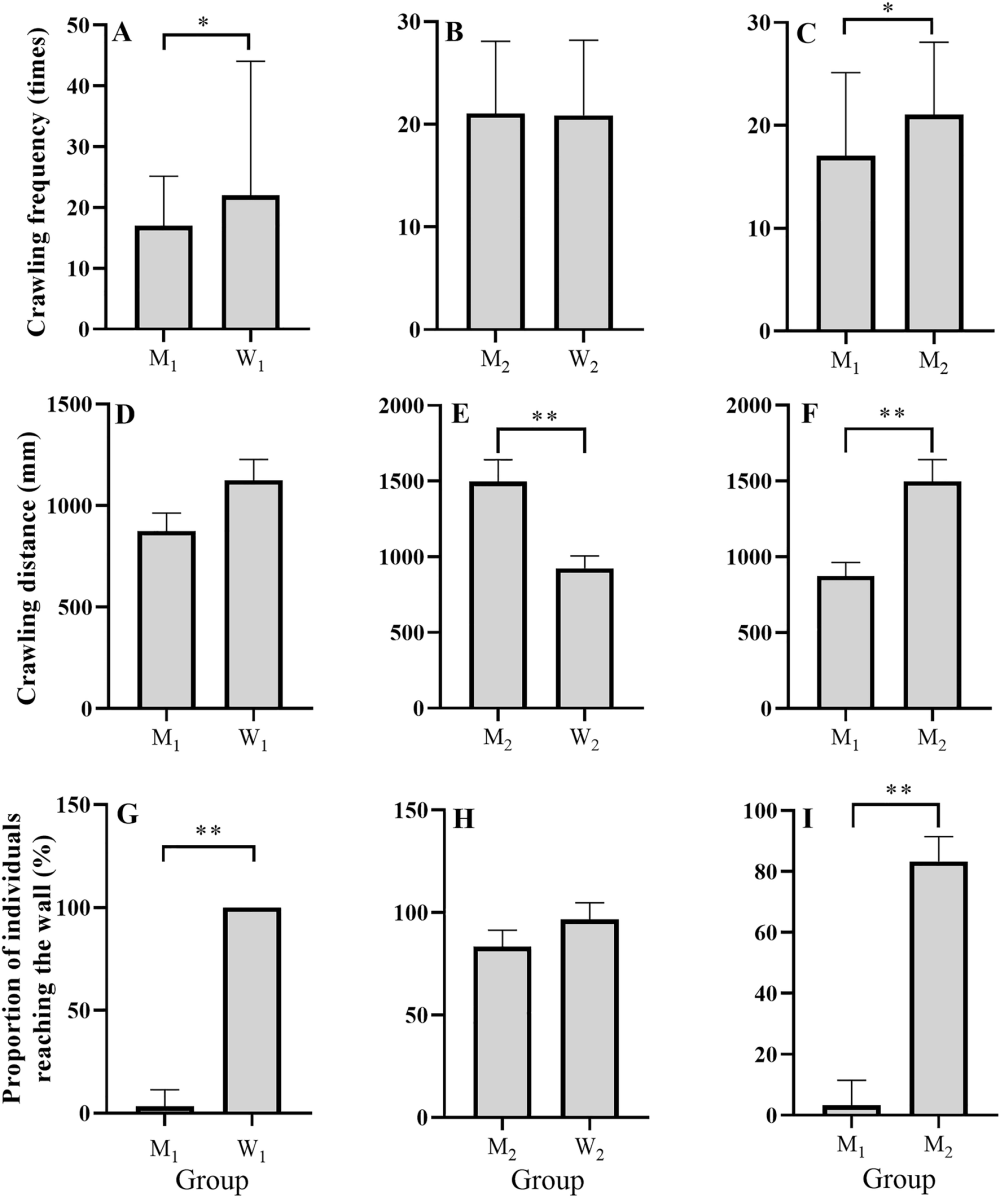
The numbers of crawling cycles in groups M_1_ and W_1_ (**A**), groups M_2_ and W_2_ (**B**), and groups M_1_ and M_2_ (**C**). Crawling distance for groups M_1_ and W_1_ (**D**), groups M_2_ and W_2_ (**E**), and groups M_1_ and M_2_ (**F**). The proportion of individuals reaching the wall for groups M_1_ and W_1_ (**G**), groups M_2_ and W_2_ (**H**), and groups M_1_ and M_2_ (**I**). Group W_1_: small seeds without mud. Group W_2_: large seeds without mud. Group M_1_: small seeds with mud. Group M_2_: large seeds with mud. The asterisks * and ** mean *P* < 0.05 and *P* < 0.01, respectively.

There was no significant difference between groups M_2_ (21.07 ± 1.28) and W_2_ (20.87 ± 1.33) in the number of crawling cycles (*t* = 0.108, *P* = 0.914) (Fig. 1B). The number of crawling cycles in group M_1_ (17.03 ± 1.48) was significantly lower than that in group W_1_ (22.03 ± 1.26) (*t* = 2.575, *P* = 0.013) (Fig. 1A) and group M_2_ (21.07 ± 1.28) (*t* = 2.060, *P* = 0.044) (Fig. 1C).

Crawling distance was not significantly different between group W_1_ (1124.13 ± 102.80 mm) and group M_1_ (874.51 ± 88.31 mm) (*t* = 1.842, *P* = 0.074) (Fig. 1D). Crawling distance in group W_2_ (923.36 ± 82.81 mm) was significantly shorter than that in group M_2_ (1498.31 ± 142.57 mm) (*t* = 3.487, *P* = 0.002) (Fig. 1E). Group M_2_ (1498.31 ± 142.57 mm) showed significantly longer crawling distance, compared to group M_1_ (874.51 ± 88.31 mm) (*t* = 3.719, *P* = 0.001) (Fig. 1F).

#### Wall-reaching behavior

No significant difference was found in the proportion of individuals reaching the wall between groups M_2_ (83.33 ± 3.33%) and W_2_ (96.67 ± 3.33%) (Mann–Whitney *U* = 30, *P* = 0.065) (Fig. 1H). Group M_1_ (3.33 ± 3.33%) showed a significantly lower proportion of individuals reaching the wall, compared to group W_1_ (100 ± 0.00%) (Mann–Whitney *U* = 36, *P* = 0.002) (Fig. 1G). The proportion of individuals reaching the wall was significantly greater in group M_2_ (83.33 ± 3.33%) than that in group M_1_ (3.33 ± 3.33%) (Mann–Whitney *U* = 36, *P* = 0.002) (Fig. 1I).

### Experiment II

#### Crawling behavior

**Figure 2:**
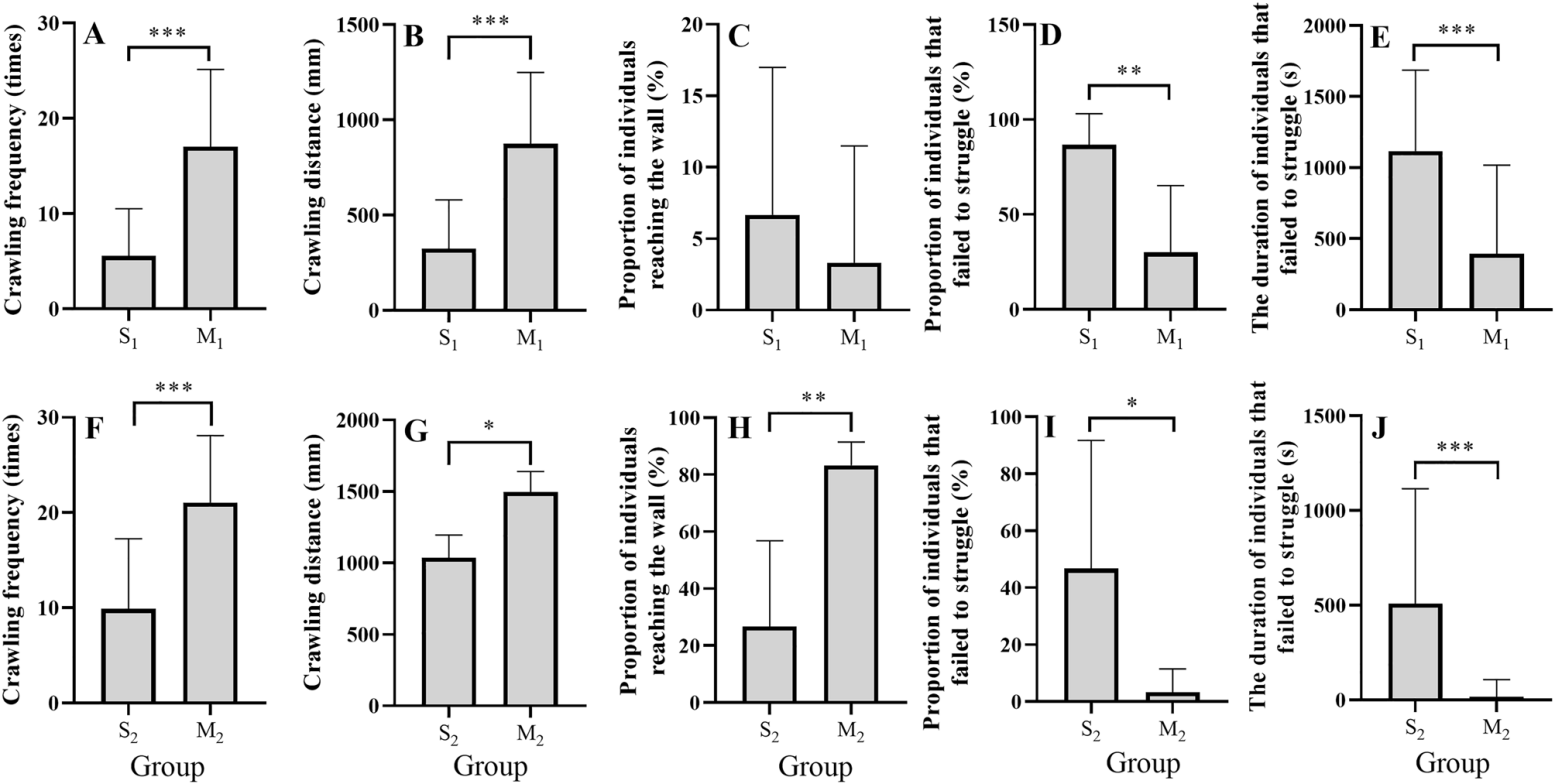
The number of crawling cycles (**A**), crawling distance (**B**), proportion of individuals reaching the wall (**C**), proportion of individuals that failed to struggle (**D**), and the duration of individuals that failed to struggle (**E**) for groups S_1_ and M_1_. The number of the crawling cycles (**F**), crawling distance (**G**), proportion of individuals reaching the wall (**H**), proportion of individuals that failed to struggle (**I**), and the duration of individuals that failed to struggle (**J**) for groups S_2_ and M_2_. Group S_1_: stressed small seeds with mud. Group S_2_: stressed large seeds with mud. Group M_1_: small seeds with mud. Group M_2_: large seeds with mud. The asterisks *, ** and ***mean *P* < 0.05, *P* < 0.01 and *P* < 0.001, respectively.

The number of crawling cycles in group S_1_ (5.57 ± 0.90) was significantly lower than that in group M_1_ (17.03 ± 1.48) (*t* = 6.610, *P* < 0.001) (Fig. 2A). Consistently, significantly lower number of crawling cycles occurred in group S_2_ (9.90 ± 1.34) than that in group M_2_ (21.07 ± 1.28) (*t* = 6.015, *P* < 0.001) (Fig. 2F).

Significantly shorter crawling distance was found in group S_1_ (324.79 ± 60.34 mm) than that in group M_1_ (874.51 ± 88.31 mm) (Mann–Whitney *U* = 34, *P* < 0.001) (Fig. 2B). Consistently, group S_2_ (1037.99 ± 157.75 mm) showed significantly shorter crawling distance than group M_2_ (1498.31 ± 142.57 mm) (Mann–Whitney *U* = 92, *P* = 0.027) (Fig. 2G).

#### Wall-reaching behavior

No significant difference was found in the proportion of individuals reaching the wall between groups S_1_ (6.67 ± 4.22%) and M_1_ (3.33 ± 3.33%) (Mann–Whitney *U* = 15, *P* = 0.699) (Fig. 2C). Group S_2_ (26.67 ± 12.29%) showed a significantly lower proportion of individuals reaching the wall, compared to group M_2_ (83.33 ± 3.33%). (Mann–Whitney *U* = 33.50, *P* = 0.009) (Fig. 2H).

#### Struggling behavior

Group S_1_ (86.67 ± 6.67%) showed a significantly greater proportion of individuals that failed to struggle than group M_1_ (30.00 ± 14.38%) (*t* = 3.576, *P* = 0.009) (Fig. 2D). Consistently, a significantly greater proportion of individuals that failed to struggle occurred in group S_2_ (46.67 ± 18.38%) than that in group M_2_ (3.33 ± 3.33%) (Mann–Whitney *U* = 7.50, *P* = 0.043) (Fig. 2I). The duration of individuals that failed to struggle was significantly longer in group S_1_ (1115.67 ± 104.19 s) than that in group M_1_ (396.40 ± 113.88 s) (*t* = 4.660, *P* < 0.001) (Fig. 2E). Consistently, significantly longer duration of individuals that failed to struggle was found in group S_2_ (508.47 ± 110.67 s) than that in group M_2_ (16.50 ± 16.50 s) (Mann–Whitney *U* = 248, *P* < 0.001) (Fig. 2J).

### Experiment III

#### Crawling behavior

**Figure 3:**
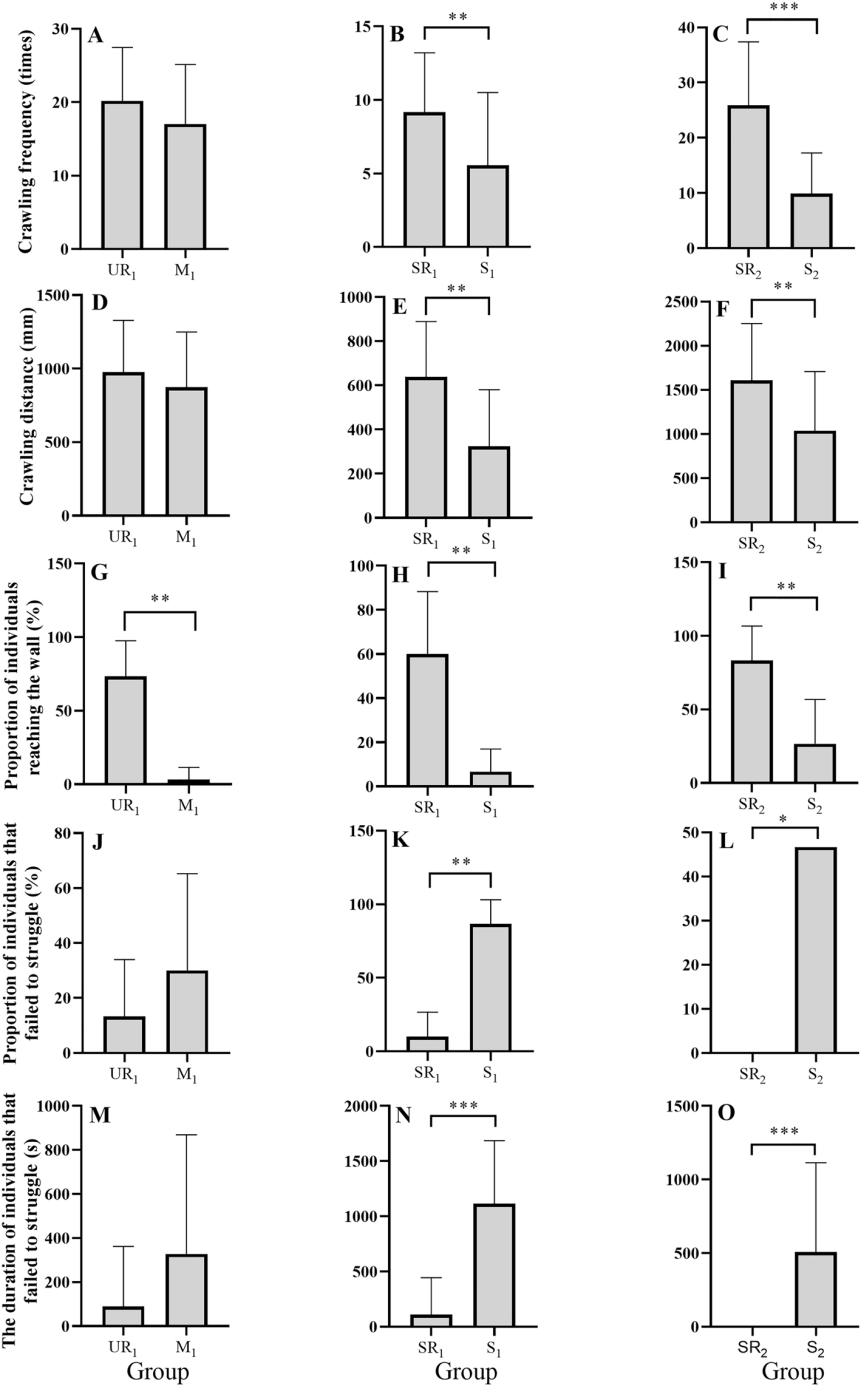
The number of crawling cycles for groups UR_1_ and M_1_ (**A**), groups SR_1_ and S_1_ (**B**), and groups SR_2_ and S_2_ (**C**). Crawling distance for groups UR_1_ and M_1_ (**D**), groups SR_1_ and S_1_ (**E**), and groups SR_2_ and S_2_ (**F**). The proportion of individuals reaching the wall for groups UR_1_ and M_1_ (**G**), groups SR_1_ and S_1_ (**H**), and groups SR_2_ and S_2_ (**I**). The proportion of individuals that failed to struggle for groups UR_1_ and M_1_ (**J**), groups SR_1_ and S_1_ (**K**), and groups SR_2_ and S_2_ (**L**). The duration of individuals that failed to struggle for groups UR_1_ and M_1_ (**M**), groups SR_1_ and S_1_ (**N**), and groups SR_2_ and S_2_ (**O**). Group S_1_: stressed small seeds with mud. Group S_2_: stressed large seeds with mud. Group M_1_: small seeds with mud. Group SR_1_: stressed small seeds with mud and artificial reefs. Group SR_2_: stressed large seeds with mud and artificial reefs. Group UR_1_: unstressed small seeds with mud and artificial reefs. The asterisks *, ** and ***mean *P* < 0.05, *P* < 0.01, *P* < 0.001, respectively.

There was no significant difference in the number of crawling cycles between groups M_1_ (17.03 ± 1.48) and UR_1_ (20.20 ± 1.33) (*t* = 1.593, *P* = 0.117) (Fig. 3A). The number of crawling cycles was significantly higher in group SR_1_ (9.17 ± 0.74 times) than that in group S_1_ (5.57 ± 0.90) (*t* = 3.087, *P* = 0.003) (Fig. 3B). The number of crawling cycles in group SR_2_ (25.87 ± 2.10) was significantly higher than that in group S_2_ (9.90 ± 1.34 times) (*t* = 6.397, *P* < 0.001) (Fig. 3C).

No significant difference was found in crawling distance between groups M_1_ (874.00 ± 88.31 mm) and UR_1_ (1033.13 ± 84.53 mm) (*t* = 0.831, *P* = 0.412) (Fig. 3D). The crawling distance in group SR_1_ (639.02 ± 59.08 mm) was significantly longer than that in group S_1_ (324.79 ± 60.33 mm) (*t* = 3.721, *P* = 0.001) (Fig. 3E). Consistently, significantly longer crawling distance occurred in group SR_2_ (1617.09 ± 170.35 mm) than that in group S_2_ (1037.99 ± 157.75 mm) (Mann–Whitney *U* = 65, *P* = 0.002) (Fig. 3F).

#### Wall-reaching behavior

The proportion of individuals reaching the wall in group M_1_ (3.33 ± 3.33%) was significantly lower than that in group UR_1_ (73.33 ± 9.8%) (Mann–Whitney *U* = 21, *P* = 0.002) (Fig. 3G). Group S_1_ (6.67 ± 4.22%) showed a significantly lower proportion of individuals reaching the wall than that in group SR_1_ (60.00 ± 11.55%) (Mann–Whitney *U* = 1.00, *P* = 0.004) (Fig. 3H). Consistently, a significantly greater proportion of individuals reaching the wall in group SR_2_ (83.33 ± 9.55%) was found than that in group S_2_ (26.67 ± 12.29%) (*t* = 3.641, *P* = 0.005) (Fig. 3I).

#### Struggling behavior

No significant difference was detected in the proportion of individuals that failed to struggle between groups M_1_ (30.00 ± 14.38%) and UR_1_ (13.33 ± 8.43%) (Mann–Whitney *U* = 23, *P* = 0.485) (Fig. 3J). Group SR_1_ (10.00 ± 6.83%) showed a significantly lower proportion of individuals that failed to struggle compared to group S_1_ (86.67 ± 6.67%) (Mann–Whitney *U* = 36, *P* = 0.002) (Fig. 3K). Consistently, a significantly lower proportion of individuals that failed to struggle occurred in group SR_2_ (0 ± 0%) than that in group S_2_ (46.67 ± 18.38%) (*t* = 2.539, *P* = 0.029) (Fig. 3L). The duration of individuals that failed to struggle in group M_1_ (327.90 ± 98.77 s) was not significantly different from that in group UR_1_ (89.70 ± 49.77 s) (Mann–Whitney *U* = 536, *P* = 0.078) (Fig. 3M). Group SR_1_ (109.27 ± 61.12 s) showed a significantly shorter duration of individuals that failed to struggle, compared to group S_1_ (1115.67 ± 104.19 s) (Mann–Whitney *U* = 807, *P* < 0.001) (Fig. 3N). Consistently, a significantly shorter duration of individuals that failed to struggle occurred in group SR_2_ (0.00 ± 0.00 s) than that in group S_2_ (508.47 ± 110.67 s) (*t* = 2.031, *P* < 0.001) (Fig. 3O).

## Discussion

### Mud negatively affects the movement-related behaviors of small *A*. *japonicus*

Improving the survival of seeds is crucial for the pond culture of sea cucumbers. In common practice, large individuals (~ 2.5 g) are seeded into the pond for better survival^15^, despite it greatly reducing the harvested net biomass and the economic benefits. There were no significant differences in the number of crawling cycles and the proportion of individuals reaching the wall of large seeds, whether they were exposed to mud or not. It has been well documented that movement capability is strongly related to the survival of sea cucumbers and sea urchins^7,16,17^. Crawling behavior is essential for sea cucumbers to move to a suitable place for feeding and habitat^18–20^. The walls of the experimental tanks simulated nearby artificial reefs in pond culture in the present study. These results suggest that mud does not negatively affect the experimental behaviors of larger seeds of sea cucumbers*.* Seeding small individuals (~ 1 g) is promising to increase economic efficiency, but it remains largely unknown why mass mortality exists when they are seeded in the ponds. This study found that the wall-reaching behavior and crawling behavior of smaller *A*. *japonicus* exposed to mud were significantly poorer than those not being exposed. Adhesion to the substrate is necessary for sea cucumbers to carry out the movement-related behaviors^13^. The mud may not have sufficient surface area for sea cucumbers to adhere^7^, which consequently reduces the crawling behavior. These results suggest that mud greatly hampers the effective movements of small *A*. *japonicus* and consequently prevents them from moving to artificial reefs, even if they are placed nearby. We further found that the crawling distance, the number of crawling cycles and the proportion of individuals reaching the wall of small seeds were significantly smaller than those of large ones, when they were both exposed to mud*.* This confirms that body size is an important factor affecting the movement-related behaviors of sea cucumbers on mud. A possible explanation is that small *A*. *japonicus* has not fully developed its motor capabilities^19^, which leads to poor performances in crawling and wall-reaching behaviors on mud. These findings explain the rationality of seeding large *A*. *japonicus* into the pond.

### Transport stress increases the adverse effects on the movement-related behaviors of *A*. *japonicus* on mud

Sea cucumber seeds are commonly transported from nurseries to ponds for further culture^4,13^, which indicates transport stress is inevitable for sea cucumbers in pond culture. The present study found that the proportion of individuals reaching the wall was significantly lower in stressed large seeds than that in the unstressed group. The significantly smaller number of crawling cycles and crawling distance consistently occurred in stressed large seeds than those in the unstressed group. These results clearly suggest that transport stress greatly reduces the ability to move in large seeds, while mud alone does not show negative effects on the behaviors of large individuals. The proportion and duration of individuals that failed to struggle consistently increased in the large seeds after they were stressed. This suggests that transport stress probably decreases the movement-related behaviors of sea cucumbers. Stressed small *A*. *japonicus* showed significantly poorer performances in the number of crawling cycles and crawling distance than those in unstressed *A*. *japonicus*. The proportion and duration of individuals that failed to struggle were significantly higher in stressed sea cucumbers than those in the unstressed group. This is consistent with the finding of Yang et al.^10^ who found that the handling stress significantly inhibited the movement and foraging behavior of small *A*. *japonicus* (~ 0.8 g). A possible explanation is that small sea cucumbers are particularly sensitive to the adverse environments^21,22^. These results collectively suggest that inevitable transport stress further increases the adverse effects on the movement-related behaviors of *A*. *japonicus* in different sizes (at least ~ 1 g and ~ 2.5 g) on mud. It is important to find a method to minimize transport stress on seeds for pond culture of sea cucumbers.

### Artificial reefs reduce the adverse effects of mud on the movement-related behaviors of *A*. *japonicus*

Developing an effective approach to reducing the adverse effects is essential to improve the production efficiency of sea cucumbers in pond culture. We found that all experimental behaviors of stressed *A*. *japonicus* seeded onto artificial reefs were significantly greater than those placed on mud. These findings suggest that artificial reefs greatly improve the movement-related behaviors of sea cucumbers and probably contribute to their survival in pond culture. Artificial reefs are commonly used to provide habitats^4,13^ and promote food utilization^23,24^ for sea cucumbers in pond culture. Seeding stressed sea cucumbers onto artificial reefs is a cost-effective method to increase the production efficiency of pond culture. Interestingly, the struggling behavior and crawling behavior of unstressed small *A*. *japonicus* did not significantly improve when they were seeded onto artificial reefs. This indicates that unstressed small *A*. *japonicus* are likely to crawl out of the artificial reefs and get stuck in the mud.

## Conclusion

There were no significant differences in the number of crawling cycles and wall-reaching behavior of large seeds exposed to mud, compared to those not exposed. Transport stress further negatively affected these movement-related behaviors of *A*. *japonicus* on mud. Seeding directly onto artificial reefs is thus an effective approach to improving the movement-related behaviors of sea cucumbers in pond culture.

Mud negatively affected the number of crawling cycles and wall-reaching behavior of small *A*. *japonicus*. Stressed small *A*. *japonicus* showed significantly poorer performances in the movement-related behaviors, compared to those unstressed on mud. Artificial reefs are beneficial to all the experimental behaviors in stressed small* A*. *japonicus*, but not in the unstressed group. We suggest that stressed sea cucumbers should be directly seeded onto artificial reefs in pond culture. Notably, the present study is a short-term experiment based on a laboratory, more evidence from long-term experiments should be collected in the field.

## Materials and methods

### Sea cucumbers

One thousand sea cucumbers of two different sizes (~ 1 g and ~ 2.5 g of wet body weight, respectively) were separately selected from a local seed hatchery and transported to Dalian Ocean University on 12 December 2021. Large (~ 2.5 g) and small (~ 1 g) seeds of sea cucumbers were maintained in fiberglass tanks (length × width × height: 1150 × 750 × 600 mm) with aeration, fed with a commercial diet (Anyuan Industrial Co., Ltd., China) ad libitum, and were under the natural photoperiod (9 L: 15 D) for two weeks until the experiment began. Water temperature was 11.08 ± 0.66 °C, salinity 32.31 ± 0.81‰ and pH 8.03 ± 0.05 according to weekly measurements (YSI Incorporated, OH, USA).

### Experiment I

**Figure 4:**
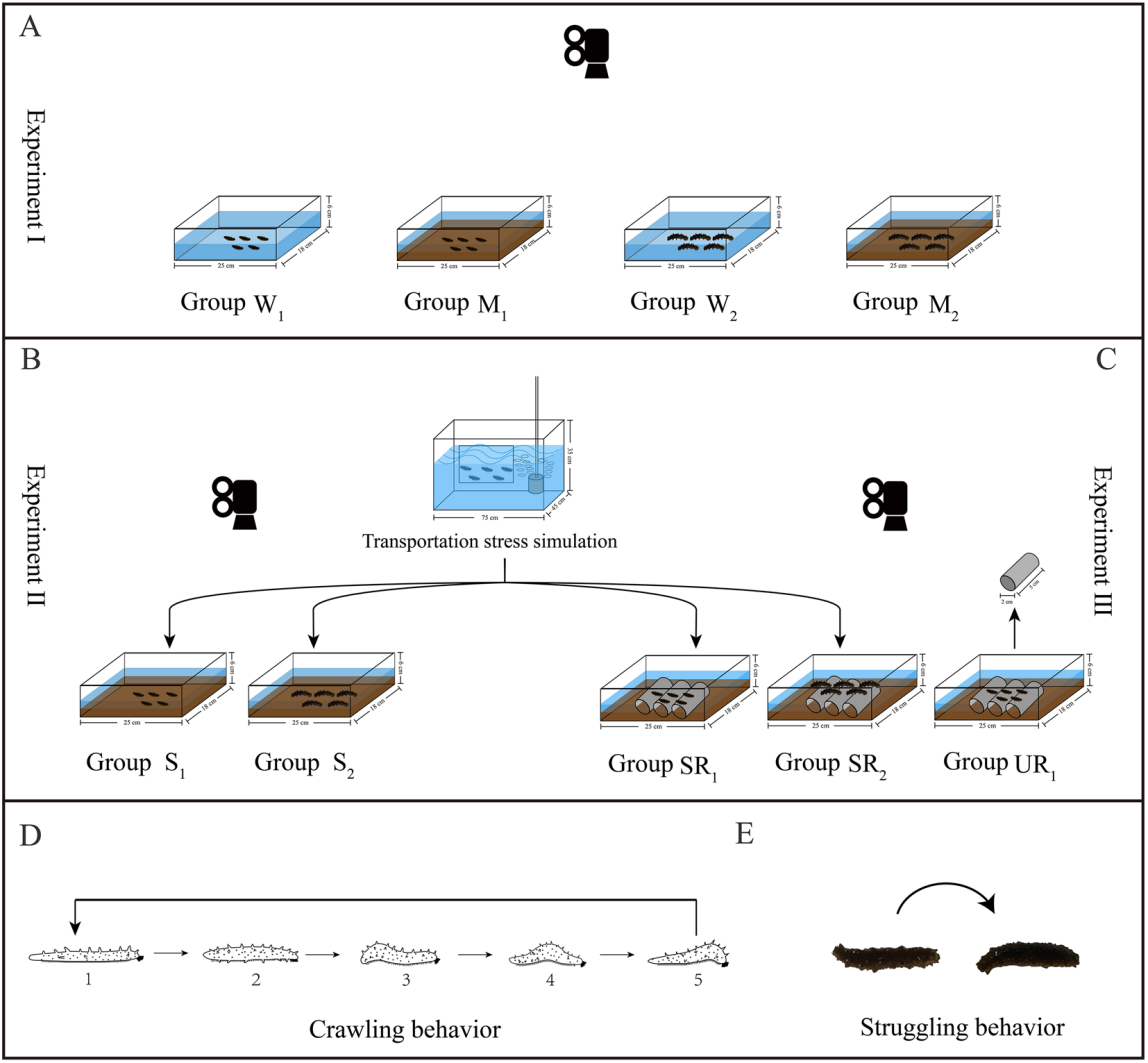
Experiment designs for experiments I (**A**), II (**B**), and III (**C**). Crawling behavior (**D**) and struggling behavior (**E**) of *Apostichopus japonicus*.

This study aimed to investigate whether mud negatively affects the movement-related behaviors of sea cucumbers in different body sizes. Mud was collected from the intertidal zone at Heishijiao (121°56′E, 38°87′N), where the mud is similar to that in pond culture. Sea mud was subsequently added to an experimental plastic tank (length × width × height: 250 × 180 × 60 mm) in a thickness of ~ 3 cm. To set groups, five sea cucumbers were placed on the bottom in the center of the experimental plastic tanks with or without mud as follows (Fig. 4A): small seeds without mud (group W_1_), large seeds without mud (group W_2_), small seeds with mud (group M_1_), and large seeds with mud (group M_2_). Each group contains six replicates (N = 6). Crawling behavior and wall-reaching behavior were subsequently evaluated according to the methods of assessment described below.

### Experiment II

This experiment was designed to investigate whether transport stress further increases adverse effects on the movement-related behaviors of *A*. *japonicus* on mud. To simulate transport stress, sea cucumbers of different body sizes were randomly selected and put into plastic bags filled with seawater in an aerated tank (length × width × height: 750 × 450 × 350 mm) for 3 h. Then, each of the five stressed sea cucumbers was subsequently placed onto the bottom and in the center of experimental plastic tanks (length × width × height: 250 × 180 × 60 mm) to set groups as follows (Fig. 4B): stressed small seeds with mud (group S_1_), and stressed large seeds with mud (group S_2_). Each group contains six replicates (N = 6). Crawling behavior, wall-reaching behavior and struggling behavior were evaluated according to the methods of assessment described below.

### Experiment III

This experiment investigated whether adverse effects can be reduced when sea cucumbers are seeded onto artificial reefs. Each of the five sea cucumbers was placed in the center of experimental plastic tanks (length × width × height: 250 × 180 × 60 mm) with mud and three connected plastic tubes (diameter × length: 20 × 50 mm, according to Tian et al.^25^ with some revisions) as follows (Fig. 4C): stressed small seeds (group SR_1_), stressed large seeds (group SR_2_) and unstressed small seeds (group UR_1_). Each group contains six replicates (N = 6). Crawling behavior, wall-reaching behavior and struggling behavior then were evaluated according to the methods of assessment described below.

### Crawling behavior

Sea cucumber crawls like earthworms to find a suitable place for feeding and habitat^7^. Crawling behavior was evaluated to assess the movement of sea cucumbers on the mud, according to Lin^26^ with some revisions. Crawling behavior was divided into five stages as follows (Fig. 4D): (1) a sea cucumber is in the inactive state; (2) the individual begins contraction; (3) the individual contracts the anus back and gradually moves by contraction of the middle of the body to the mouth; (4) the individual gradually stops contraction and (5) returns to the inactive state. Crawling behavior was recorded for 30 min using a digital camera (Legria HF20, Canon, Japan). One crawling cycle covers the above five stages. The crawling distance was measured using the software Image J (version 1.51n).

### Wall-reaching behavior

Sea cucumbers tend to move until they contact the wall of the aquarium and remain there^7^. This behavior simulated the situation in that sea cucumbers get rid of the mud and successfully move to nearby artificial reefs in the pond. A digital camera (Legria HF20, Canon, Japan) was used to record the behaviors for 30 min and then the collected data was used to calculate the proportion of sea cucumbers reaching the wall.

### Struggling behavior

Struggling behavior was evaluated based on Clements et al.^27^ with some revisions. Sea cucumbers lose their ability to move normally on the mud^7^, and they try to correct their body posture. If fail to struggle, they may get stuck in the mud and consequently die. The struggling behavior of sea cucumbers on mud was recorded for 30 min using a digital camera (Legria HF20, Canon, Japan) (Fig. 4E). We calculated the proportion and duration of individuals that failed to struggle based on the collected data by using the camera.

### Statistical analysis

Kolmogorov–Smirnov test and Levene test were used to analyze the distribution and homogeneity of variance, respectively. The independent-sample *t* test was used to compare the differences in the number of crawling cycles and the crawling distance in experiment I; the number of crawling cycles, the proportion and duration of individuals that failed to struggle (both between groups M_1_ and S_1_) in experiment II; the number of crawling cycles and the crawling distance (except between groups SR_2_ and S_2_), the proportion of individuals reaching the wall (between groups SR_2_ and S_2_), the proportion and duration of individuals that failed to struggle (both between groups SR_2_ and S_2_) in experiment III. Mann–Whitney *U* test was carried out to analyze the rest of the data, because of non-normal distribution and/or heterogeneity of variance. All data analyses were performed using SPSS 19.0 statistical software. A probability level of *P* < 0.05 was considered as being significant.

### Ethical approval

All applicable international, national, and/or institutional guidelines for the care and use of animals were followed by the authors.
